# Uncertain threat is associated with greater impulsive actions and neural dissimilarity to Black versus White faces

**DOI:** 10.3758/s13415-022-01056-2

**Published:** 2023-02-02

**Authors:** Estée Rubien-Thomas, Nia Berrian, Kristina M. Rapuano, Lena J. Skalaban, Alessandra Cervera, Binyam Nardos, Alexandra O. Cohen, Ariel Lowrey, Natalie M. Daumeyer, Richard Watts, Nicholas P. Camp, Brent L. Hughes, Jennifer L. Eberhardt, Kim A. Taylor-Thompson, Damien A. Fair, Jennifer A. Richeson, B. J. Casey

**Affiliations:** 1 Department of Psychology, Yale University, New Haven, CT USA; 2 Pritzker School of Medicine, University of Chicago, Chicago, IL USA; 3 Department of Psychology, Temple University, Philadelphia, PA USA; 4 Columbia University College of Physicians and Surgeons, New York, NY USA; 5 Departments of Occupational Therapy and Neurology, Washington University School of Medicine in St. Louis, St. Louis, MO USA; 6 Department of Psychology, Emory University, Atlanta, GA USA; 7 Department of Organizational Studies, University of Michigan, Ann Arbor, MI USA; 8 Department of Psychology, University of California Riverside, Riverside, CA USA; 9 Department of Psychology, Stanford University, Stanford, CA USA; 10 School of Law, New York University, New York, NY USA; 11 Masonic Institute for the Developing Brain, Minneapolis, MN USA; 12 Department of Neuroscience and Behavior, Barnard College, New York, NY USA

**Keywords:** Brain, Cognitive control, Implicit bias, Race, Threat, Uncertainty

## Abstract

Race is a social construct that contributes to group membership and heightens emotional arousal in intergroup contexts. Little is known about how emotional arousal, specifically uncertain threat, influences behavior and brain processes in response to race information. We investigated the effects of experimentally manipulated uncertain threat on impulsive actions to Black versus White faces in a community sample (n = 106) of Black and White adults. While undergoing fMRI, participants performed an emotional go/no-go task under three conditions of uncertainty: 1) anticipation of an uncertain threat (i.e., unpredictable loud aversive sound); 2) anticipation of an uncertain reward (i.e., unpredictable receipt of money); and 3) no anticipation of an uncertain event. Representational similarity analysis was used to examine the neural representations of race information across functional brain networks between conditions of uncertainty. Participants—regardless of their own race—showed greater impulsivity and neural dissimilarity in response to Black versus White faces across all functional brain networks in conditions of uncertain threat relative to other conditions. This pattern of greater neural dissimilarity under threat was enhanced in individuals with high implicit racial bias. Our results illustrate the distinct and important influence of uncertain threat on global differentiation in how race information is represented in the brain, which may contribute to racially biased behavior.

**Supplementary Information:**

The online version contains supplementary material available at 10.3758/s13415-022-01056-2.

## Introduction

Race remains an evocative, visual indicator of group membership that can impact emotional arousal, cognition, and behavior. Despite a reduction in explicit endorsement of many racial stereotypes over the past few decades in the United States (Devine & Elliot, 1995; Madon & Palumbo, 2001; but see Bobo et al., 2012), implicit activation of racial stereotypes and biased beliefs persist in more subtle ways (Nosek & Greenwald, 2002). Implicitly held racial stereotypes are thought to reflect, at least in part, culturally shared beliefs about racial groups imbued within society-at-large (Payne et al., 2017) and can contribute to behavior through a wide variety of processes, including attention and cognitive control (Correll et al., 2002; Dickter & Bartholow, 2017; Rubien-Thomas et al., 2021; Trawalter et al., 2008). Foundational neuroimaging work has identified key brain circuits that respond to information about race and contribute to subsequent behavior toward people based on race. However, much remains to be understood regarding the patterns on race-based neural activity observed in past work. Furthermore, because the majority of studies examining the effects of racial category information on brain responses have been conducted with predominantly White samples (Correll et al., 2002; Natu et al., 2011; Rubien-Thomas et al., 2021), less is known about how an individuals’ own racial group membership may contributes to the neural representation of race information. Building on past work, the current study investigated the contributions of uncertain threat (i.e., anticipation of an unpredictable negative event) and implicit bias in shaping impulsive actions and spatial patterns of brain activity in response to racial category information in a community sample of Black and White individuals adults.

Uncertain threat is the anticipation of an unpredictable aversive event, regardless of whether imminent danger is actually present (Barlow, 2000; Weiss, 1970). Uncertain threat differs from certain threat (predictable threat) by the unpredictability of if and when an aversive cue will occur (Davis et al. 2010; Davies & Craske, 2015). It is evolutionarily adaptive for our survival to identify potential threats quickly. For example, biologically threatening stimuli (e.g., spiders, bears) rapidly capture our attention (Ohman et al., 2001). Likewise, threat-related faces (e.g., perceived untrustworthiness) are processed rapidly and automatically (Freeman 2014; Chua & Freeman, 2021) and are historically interpreted as an evolutionarily adaptive or innate mechanism important for survival (Oosterhof & Todorov, 2008). However, there is growing evidence that experiences play an important role in the formation of these perceptions (Chua & Freeman, 2021; Dotsch et al., 2016; Stolier et al., 2020).

The perception of uncertain threat may be especially relevant to the processing of racial category information. Specifically, the belief that Black individuals are dangerous and threatening is a shared cultural stereotype in the United States that persists to this day (Cottrell & Neuberg, 2005; Duncan, 1976; Eberhardt et al., 2004; Gilbert, 1951; Xie et al., 2021). The faces of Black individuals have been shown to induce heightened arousal (Blascovich et al., 2001; Mendes et al., 2002), activate fear-related circuitry (Cunningham et al., 2004, 2008; Hart et al., 2000; Lieberman et al., 2005; Phelps et al., 2000; Richeson et al., 2008), and enhance attentional bias (Bean et al., 2012; Donders et al., 2008; Otten, 2016; Trawalter et al., 2008) in predominantly White samples. The strength of the perceivers’ racial biases moderates activation of fear-related circuitry and enhanced attentional pull to Black faces (Donders et al., 2008; Hatzenbuehler et al., 2021; Krill & Platek, 2009; Phelps et al., 2000). Together this work implicates uncertain threat in the patterns of behavior, physiological arousal, and neural activity in response to Black faces. Yet to date, uncertain threat has not been experimentally manipulated when examining behavioral and brain responses to Black faces.

Previous work has demonstrated the influence of racial category information on multiple neural and cognitive processes. Visual areas, including the fusiform gyrus, show sensitivity to social identities such as race in both the magnitude and spatial patterns of blood oxygenation level-dependent (BOLD) activity in the brain (Contreras et al., 2013; Golby et al., 2001; Natu et al., 2011; Rubien-Thomas et al., 2021). Activity of the fusiform in response to racial as well as other social group information appears to be moderated by relevant racial stereotype information (Xie et al., 2021) with endorsement of social group stereotypes being associated with greater sensitivity in the fusiform gyrus to faces of individuals in that social group (Brosch et al., 2013; Xie et al., 2021). Furthermore, racial stereotypes are associated with altered functional connectivity of this region with higher-order frontoparietal and default mode networks (Barnett et al., 2021), suggesting that racial stereotypes not only influence the representation of race information in the visual cortex but also influence a broader range of interconnected prefrontal networks. Moreover, altered activity in frontoparietal networks (Cassidy & Krendl, 2016; Richeson et al., 2003; Rubien-Thomas et al., 2021) and changes in functional connectivity between visual and frontoparietal brain regions (Barnett et al., 2021; Brown et al., 2017) in response to race information have been associated with diminished cognitive control to Black faces (Brown et al., 2017; Correll et al., 2002; Richeson et al., 2003; Richeson & Shelton, 2003; Richeson & Trawalter, 2005; Rubien-Thomas et al., 2021). This brain-behavior association also has been attributed to threat-related processes, including negative stereotypes of Black individuals (Correll et al., 2007; Rubien-Thomas et al., 2021) and to the social threat of appearing prejudiced (Amodio et al., 2008; Richeson & Trawalter, 2008). As a whole, the literature provides evidence of multiple brain networks contributing independently and together when processing or reacting to salient race-relevant information (Amodio & Cikara, 2021; Kubota, Banaji, and Phelps 2012). Moreover, this emergent body of research triangulates on uncertain threat as a critical factor in shaping brain and behavioral responses to Black compared with White individuals.

The current study tests the relevance, if not centrality, of uncertain threat in the processing of race. We utilized a psychophysiologically validated (Cohen et al., 2016) impulse control paradigm in which race information is irrelevant to the task instructions (i.e., detection of emotional faces as targets or nontargets regardless of race) and compared behavioral and brain responses to race cues (Black and White faces) under experimentally manipulated states of emotional arousal and uncertainty that include: 1) when anticipating an uncertain threat; 2) when anticipating an uncertain reward; and 3) when anticipating no event. We hypothesized that participants would impulsively react to Black faces relative to White faces under the condition of uncertain threat more than in comparison conditions based on the aforementioned literature showing diminished cognitive control to Black cues (Correll et al., 2002; Richeson et al., 2003; Rubien-Thomas et al., 2021) and linking it to the perceptions of threat. To test our hypothesis, we examined differences in impulse control in response to an equal number of Black and White face stimuli from validated, open-access diverse face stimuli sets (Conley et al., 2018; Tottenham et al., 2009) under each of the three uncertainty conditions. Unlike many behavioral and brain imaging studies examining the impact of race information on behavior in predominantly White participants, we tested a community sample with equal representation of Black and White participants. To the extent that Black participants respond to Black, compared with White, faces in a manner that is similar to White participants, we will have even stronger evidence for the critical role of uncertain threat—rather than outgroup derogation or animus—in the emergence of patterns of cognitive control failure in response to Black faces found in previous work. We then used representational similarity analysis (RSA; Kriegeskorte et al., 2008) to examine dissimilarity in neural representations of Black and White faces under conditions of uncertainty across functional brain networks. Finally, to constrain the interpretation of our results, we examined whether scores on a measure of implicit racial bias (Implicit Association Test, Greenwald et al., 1998) differentially contributed to neural dissimilarity in representation of Black and White faces under different conditions of uncertainty.

## Methods

### Sample

A community sample of 106 Black and White, healthy, right-handed adults (18-37 years, mean [standard deviation (SD)] age = 26.0, [5.2] years; 53% Black, 53% female) was recruited from the greater New York City, New York, and New Haven, Connecticut, metropolitan areas. Data from 1 Black participant was excluded due to scan parameter inconsistencies during data acquisition, resulting in a sample of 105. Six participants (4 Black, 2 females) had invalid IAT scores based on recommended reaction time exclusion criteria (>10% of trials with latencies <300 ms) (Greenwald & Nosek, 2003) and were excluded from all IAT analyses. No participants reported any previous or current diagnoses of psychiatric or neurological disorders or use of psychotropic medications. All participants provided written consent approved by institutional review boards at their respective data collection sites.

### Emotional go/no-go task

**Fig. 1:**
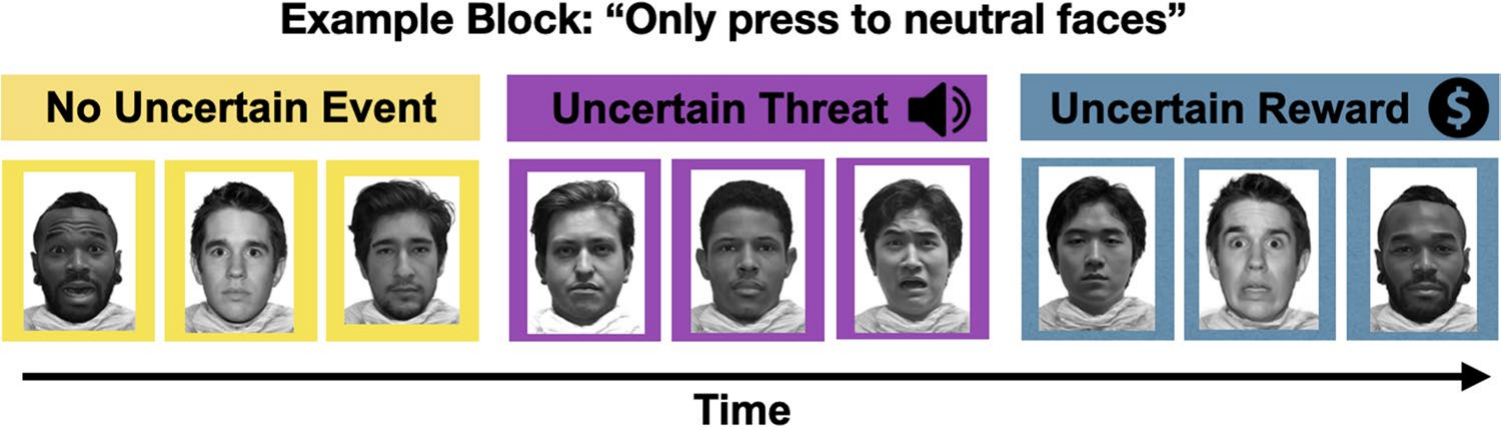
Experimental paradigm of the emotional go/no-go task. Trials are displayed within blocks with no anticipation of an event (no uncertain event), negative arousal in anticipation of an unpredictable aversive noise (uncertain threat), and positive arousal in anticipation of an unpredictable monetary reward (uncertain reward). Blocked experimental conditions are indicated by the background color of the screen which were counterbalanced across participants

A modified, emotional, go/no-go task was used (Cohen et al., 2016) that includes the brief presentation of fearful, happy, and neutral male faces as both targets and nontargets under three conditions of uncertainty: 1) anticipation of threat (i.e., unpredictable loud aversive sound and negatively valenced picture); 2) anticipation of reward (i.e., unpredictable receipt of money); and 3) no anticipation of an uncertain outcome. During uncertain threat, participants were instructed that an unpredictable negative event may occur (possibility of aversive auditory stimulus paired with an image of a snarling dog). During uncertain reward, participants were instructed that an unpredictable positive event may occur (possibility of winning up to $100 paired with an image of cash and sound of a slot machine). In a third condition, participants were instructed that no event would occur (no possibility of the negative or positive event occurring). Images for the uncertain threat and reward conditions were selected from the International Affective Picture System and matched for similar arousal ratings (Lang et al., 1997). Conditions of uncertainty were indicated by the background color (yellow, purple, or blue) of the screen during the task (Fig. 1), which were counterbalanced across participants. Arousal during the uncertain threat and reward conditions has been validated previously with psychophysiological arousal measures (galvanic skin conductance) and subjective ratings (Cohen et al., 2016).

Participants were instructed that the probability and timing of a threat or reward during its respective color block was uncertain (determined randomly by the computer) and was not dependent on their task performance. In actuality, each participant heard the aversive noise and received money ($20) once during the task, each toward the end of a run in a pseudo-randomized order so that these periods could be removed easily from imaging analyses. Participants were instructed to press a button as quickly as they could for targets (“go” trials) and withhold a response for nontargets (“no-go” trials). At the beginning of each run, participants were told what emotional face was the target (“go” cue). The stimulus duration of each trial was 500 milliseconds, followed by a jittered intertrial interval of 2-8 seconds. A total of 102 trials were presented in a pseudo-randomized order within each run (72 go trials, 30 no-go trials).

The fMRI data were acquired in six 7-minute runs. Together, all runs consisted of every combination of the negative, positive, and neutral faces as both a target and nontarget. Each condition of uncertainty (uncertain threat, uncertain reward, no uncertain event) occurred twice as blocks within a run. Pairing of background color with instructed state of uncertainty was counterbalanced among participants and run orders were pseudo-counterbalanced. Male face stimuli for the go/no-go task were selected from previously validated sets of emotional face stimuli (Conley et al., 2018; Tottenham et al., 2009). To test for differences in responses to Black versus White faces, the task was optimized to include 45% White faces, 45% Black faces, and 10% Asian and Hispanic “foil” faces in an attempt to obscure the significance of race in the task and to control for a potential novelty effect of a less frequently presented race, which were equally distributed across each experimental condition. Participants completed a practice session of the task before starting the session to ensure that they understood the task conditions and instructions. In sum, the present task allows for the comparison of responses to Black versus White faces under a state of uncertain threat relative to nonarousing and positively arousing comparison conditions.

### Implicit association test

Participants completed a well-validated measure of implicit racial bias (Greenwald et al., 1998), in which individuals sort photographs of the faces of Black and White Americans and positive and negative words into stereotype-congruent (Black/Bad & White/Good) and stereotype-incongruent (Black/Good & White/Bad) blocks of trials (2 test blocks of 40 trials used for scoring). The IAT was completed at the end of the study in an effort to minimize an experimental emphasis on race and influence on emotional go/no-go task performance.

### fMRI data acquisition

Sequence parameters were based on previously published ABCD imaging parameters (Casey et al., 2018). Images were acquired on Siemens Prisma 3T scanners at both sites (version VE11B). Anatomical images were acquired using a T1-weighted sequence (repetition time [TR] = 2,400 ms; echo time [TE] = 2.12 ms; T1 = 1,060 ms; flip angle, 8 degrees; 256 × 256 matrix; sagittal slices, 208; resolution, 1 mm^3^) and a T2-weighted sequence (repetition time [TR] = 3,200 ms; echo time [TE] = 564 ms; 256 × 256 matrix; sagittal slices, 208; resolution, 1 mm^3^). Whole-brain, echo-planar imaging (EPI) volumes were acquired with T2*-weighted EPI sequence sensitive to the blood oxygenation level dependent (BOLD) contrast (TR = 800 ms; TE = 30.0 ms; flip angle, 49 degrees; voxel size 2.4 mm^3^; AC-PC oriented slices, 66). MRI data were acquired by using the same imaging parameters at both sites.

### Behavioral analyses

False-alarm rates (i.e., percent of incorrect no-go trials) were the primary dependent measure of impulse control. Because our primary hypothesis centered on the effects of uncertain threat and limited power to test for interactions of emotional cues and uncertainty conditions, false-alarm rates to Black faces and White faces were calculated within each uncertainty condition (collapsed across emotional cues). To confirm that emotional cues did not significantly contribute to model fit, we show that a linear model without a predictor of emotional cue (R_marginal_^2^ = 0.11) results in a significantly better fit than a model including emotional cues (R_marginal_^2^ = 0.060, 𝜒^2^(24) = 224.53, *p* < 0.001). All statistical analyses were conducted by using R (R Core Team, 2021), and the lme4 package (Bates et al., 2015) was used to perform linear mixed-effects analyses, where subjects were treated as a random effect nested within site (i.e., random intercepts for each participant). Accounting for the magnitude of effects at the group level was of particular interest; thus fitting a maximal model was attempted, but random slopes needed to be removed for the model to converge, leaving only intercepts as random parameters for each participant. A linear mixed-effect model with maximum likelihood estimates of parameters was used to determine the effects of stimulus race and uncertainty condition on false alarm rates and compared the model with a null model (Faraway, 2006). False-alarm rates were mean-centered so that y-intercepts reflected the grand mean of the dependent variable (Afshartous & Preston, 2011). Between-subject factors of participant race and participant gender, and within-subject factors of stimulus race and uncertainty condition, were treated as fixed effects with interaction terms between participant race, stimulus race, and uncertainty condition (see Equation 1). All models were calculated to optimize log-likelihood criterions. Visual inspection of all residual plots revealed normal distributions for each model with no obvious deviations from homoscedasticity or normality. Full models were compared with the appropriate null model by using likelihood ratio tests. Satterthwaite’s method was used for obtaining degrees of freedom and *p*-values for linear mixed models (Luke, 2017; Satterthwaite, 1946).

1 $${\displaystyle \begin{array}{c}\textrm{False}\ \textrm{Alarm}\ \textrm{Rate}={\beta}_1\cdotp \textrm{Stimulus}\ \textrm{Race}\ \textrm{x}\kern0.5em {\beta}_2\cdotp \textrm{Uncertainty}\ \textrm{Condition}\ \textrm{x}\\ {}{\beta}_3\cdotp \textrm{Participant}\ \textrm{Race}+{\beta}_4\cdotp \textrm{Participant}\ \textrm{Gender}+{\upeta}_{\left(\textrm{Site}\ |\ \textrm{Subject}\right)}+e\ \end{array}}$$

Scoring of the Implicit Association Test followed the algorithm recommended by (Greenwald & Nosek, 2003). D-scores were calculated for each participant as a standardized measure of the relative strength of positive and negative associations with Black faces. Positive scores indicate pro-White bias and negative scores indicate pro-Black bias. Six participants (4 Black, 2 White) had invalid scores (>10% of trials had response times <300 ms) based on the Greenwald et al. (2003) scoring algorithm.

### fMRI data analysis

Structural and functional imaging data were preprocessed using the Human Connectome Project (HCP) Minimal Preprocessing Pipeline version 3.17 for image correction, localization, and registration (described in detail in Glasser et al., 2013). Specifically, functional images were corrected for gradient distortions by using spin echo field maps with opposite phase encoding directions, intensity normalized to the grand-mean, corrected for head motion, and transformed to standard space (MNI 152, 2-mm voxels) using nonlinear registration. Volumetric outputs from the fMRI processing step in the HCP Minimal Preprocessing Pipeline were used for analyses. FSL (FMRIB Software Library (Woolrich et al., 2001) was used for neuroimaging analyses.

General linear models (GLMs) were run for each participant and included three block regressors representing the three conditions of uncertainty and 12 event-related regressors representing each trial type within each uncertainty condition for 3 race conditions [Black, White, foil] x 4 trial types [correct go; correct no-go; incorrect go; incorrect no-go]), resulting in 39 first-level regressors. Each event-related trial was modeled for 500 milliseconds, corresponding to the duration of the stimulus presentation and convolved with a double-gamma hemodynamic response function. Temporal derivatives of each stimulus predictor were included as additional regressors. Time series data were prewhitened to account for autocorrelations. Six additional regressors were included in the GLM to account for head movement (x-, y-, and z-translations and rotations). Volumes with greater than 0.9-mm framewise displacement were excluded from analyses, as well the neighboring volumes (Power et al., 2014). First-level analyses were smoothed at 5-mm, full-width, half-maximum of the Gaussian kernel. Second-level analyses calculated mean response across runs of the task for each participant.

### Representational similarity analysis

We next asked how the spatial representation of Black and White faces differed in the brain across the three experimental conditions. We approached this question by using representational similarity analysis (RSA), which is a widely used multivariate voxel-wise pattern analysis (MVPA) method to examine similarity (or dissimilarity) of the spatial patterns of BOLD response between experimental conditions (Kriegeskorte et al., 2008). In our primary analyses, we used RSA to evaluate the dissimilarity in spatial patterns of brain activity to Black and White faces in each uncertainty condition.

More specifically, for each participant and for each pair of experimental conditions (certainty condition [uncertain threat/uncertain reward/no uncertain event] * stimulus race [White/Black]), the correlation distance between each experimental condition was calculated within functionally defined brain regions using whole-brain t-statistic maps of go trials as inputs. Statistics maps of go trials were used because of their relative frequency within the task, therefore maximizing the signal in our input for calculating correlation distance. Within each given brain region, spatial similarity was computed by using Pearson’s correlation. Those values were then subtracted from 1 (1-Pearson r), resulting in a representational dissimilarity matrix (RDM) of all pairwise combinations of uncertainty conditions. This process was repeated for each of 300 distinct functional regions (Schaefer et al., 2018) for each participant. Each region in the Schaefer et al. (2018) parcellation is associated with one of seven functional networks previously defined by Yeo et al. (2011): frontoparietal, default, dorsal attention, limbic, salience/ventral attention, somatomotor, visual. To assess the effects of uncertainty on the neural representations of race at the level of functional networks, RDM correlation distance values were averaged across regions in a given functional network for each participant.

Paralleling our behavioral model, we next sought to determine the effects of stimulus race and uncertainty condition on the representational dissimilarity between Black and White faces across all participants. To do so, we performed a network-level analysis where correlation distance values were submitted to a linear mixed-effects model. Conditions of uncertainty and the functional networks were treated as within-subject fixed effects (see Equation 2). Participant race was not included in the model, because it did not show a main effect or three-way interaction with our factors of interest (stimulus race and uncertainty condition) in the behavioral model.

2 $$\textrm{Correlation}\ \textrm{Distance}={\upbeta}_1\cdotp \textrm{Uncertainty}\ \textrm{Condition}\ \textrm{x}\kern0.5em {\upbeta}_2\cdotp \textrm{Functional}\ \textrm{Network}+{\upeta}_{\left(\textrm{Site}\ |\ \textrm{Subject}\right)}+e$$

A secondary analysis was conducted to test for the potential contributions of implicit bias on the spatial representation of race in the brain under the anticipation of threat and reward. In this analysis, a mixed model was performed with the uncertainty conditions, functional networks, and implicit bias as fixed effects with interaction terms between each term. For both primary and secondary imaging analyses, subjects were treated as a random effect nested within site (i.e., random intercepts for each participant). Models were conducted with maximum likelihood estimates of parameters to compare with a null model (Faraway, 2006) and Satterthwaite’s method (Luke, 2017; Satterthwaite, 1946) to obtain degrees of freedom and *p*-values.

## Results

### Behavioral results

**Fig. 2:**
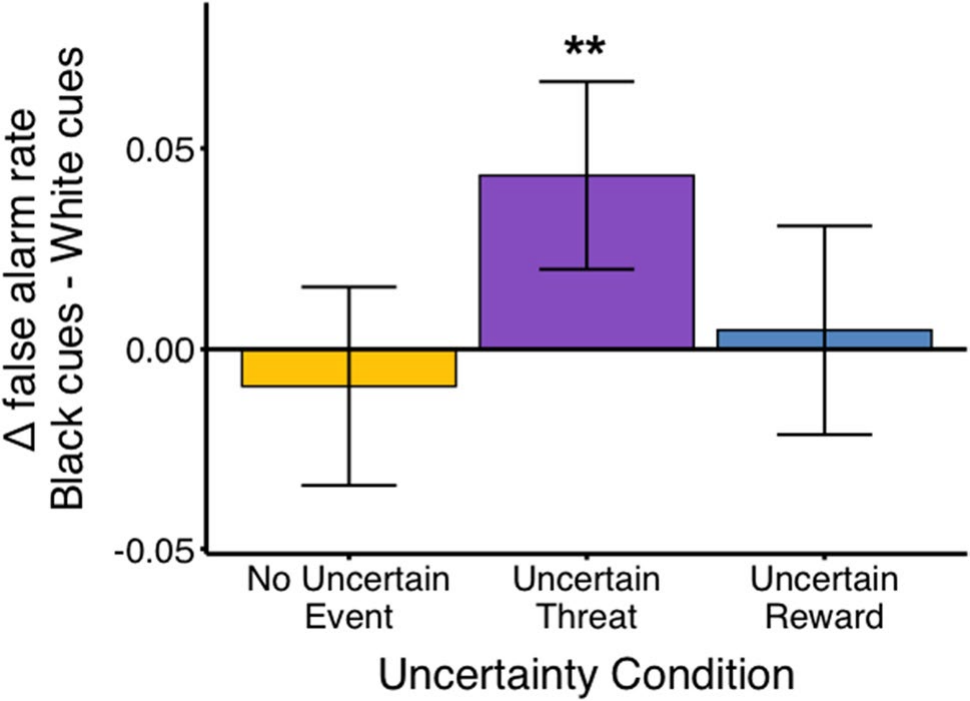
Participants, regardless of their own race, show increased impulsive actions to Black compared with White faces when anticipating uncertain threat. Error bars represent 95% confidence intervals

A linear mixed-effect model revealed significant main effects of uncertainty condition (*F*(2, 525.00) = 9.11, *p* < 0.001), gender (*F*(1, 104.53) = 9.79, *p* = 0.0023), an interaction between participant race x stimulus race (*F*(1, 525.00) = 8.77, *p* = 0.0032; Figure S1) and consistent with our hypothesis, an interaction between stimulus race and uncertainty condition on false-alarm rates (*F*(2, 525.00) = 4.14, *p* = 0.016) where only in the uncertain threat condition were false alarms greater to Black faces compared with White faces (Table S2). No main effect of participant race was observed (*F*(1, 104.65) = 0.065, *p* = 0.80). Likelihood ratio testing comparing the full model against a model excluding the interaction term of interest (uncertainty condition x stimulus race) revealed a significant difference (𝜒^2^(4) = 10.00, *p* = 0.040), with the full model accounting for greater variation (R_marginal_^2^ = 0.082) than the null model (R_marginal_^2^ = 0.079; Table S1). To summarize and visualize the relevant comparisons (uncertainty condition x stimulus race), difference scores in false-alarm rates to Black and White faces within an uncertainty condition were calculated. Individuals, regardless of their own race, showed higher false alarm rates to Black compared with White faces (as evidenced by positive difference scores) when anticipating an uncertain threat (one-tailed *t*-test compared with 0, *t*(104) = 3.1, *p* = 0.002; Fig. 2). Difference scores did not differ from zero in the uncertain reward and no uncertain event conditions (one-tailed *t*-test compared with 0, *p*’s > 0.50). In other words, individuals, regardless of their own race, had significantly higher false-alarm rates to Black compared with White faces when anticipating uncertain threat. A Pearson’s correlation analysis indicated that IAT scores were not associated with difference scores in false-alarm rates to Black and White faces in any of the three uncertainty conditions (*p*’s > 0.79).

### Implicit association test results

Although the distribution of IAT scores overlapped for Black and White participants, White participants (*M*_White_ = 0.55 , *SD*_White_ = 0.52) showed significantly higher (more pro-White) implicit bias scores than Black participants (*M*_Black_ = −0.053 , *SD*_Black_ = 0.48; (*t*(95.15 = 6.01, *p* < 0.001; Figure S2.)

### Imaging results

**Fig. 3:**
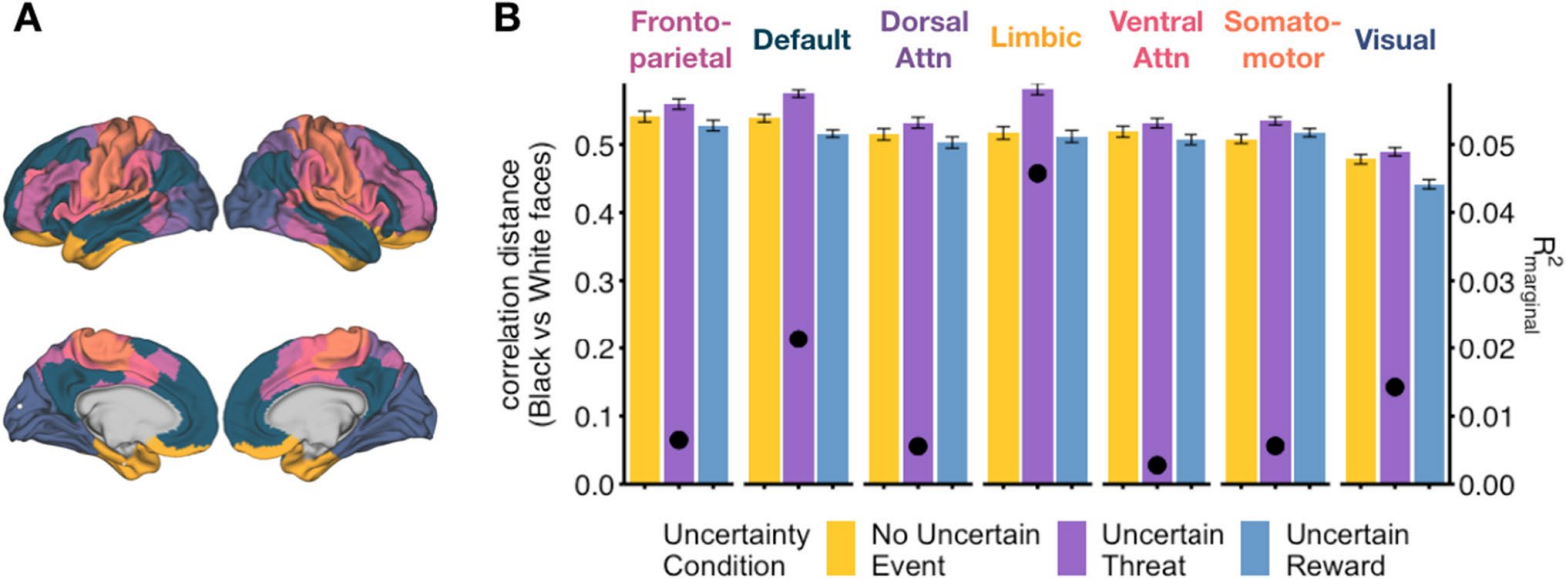
Neural dissimilarity to Black versus White faces by uncertainty condition. **A.** Parcellation of seven functional networks represented by different colors. **B.** Neural dissimilarity is greatest in the uncertain threat condition compared to the no event and uncertain reward conditions across all seven networks. Left axis and bars depict the correlation distance between Black and White faces for each uncertainty condition in each functional network. Right axis and Black dots represent the effect size of the uncertainty condition on correlation. Error bars represent 95% confidence intervals

A linear mixed-effects model revealed main effects of uncertainty condition (*F*(2, 1980.00) = 13.51, *p* < 0.001) and network (*F*(6, 1980.00) = 8.02, *p* < 0.001) in predicting the dissimilarity between Black and White faces. Likelihood ratio testing comparing the full model against a model without the factor of interest (uncertainty condition) revealed a significant difference (𝜒^2^(14) = 34.82, *p* = 0.0016), with the full model minimizing information criteria to a greater extent (R_marginal_^2^ = 0.032) than the null model (R_marginal_^2^ = 0.019, Table S3). Correlation distances between Black and White faces under uncertain threat were significantly higher than those in the no uncertain event (paired *t*-test, *t*(692) = 3.14, *p* = 0.0018) and uncertain reward conditions (*t*(692) = 4.17, *p* < 0.001) (Fig. 3). In other words, the neural dissimilarity between Black and White faces, regardless of functional network, was greatest in anticipation of uncertain threat. Limbic (which includes threat related circuitry), default, and visual (which includes the fusiform gyrus) networks showed the greatest effect of uncertainty condition (R_marginal_^2^ > 0.15), with other networks demonstrating significant but smaller effect sizes (R_marginal_^2^ < 0.0065).

**Fig. 4:**
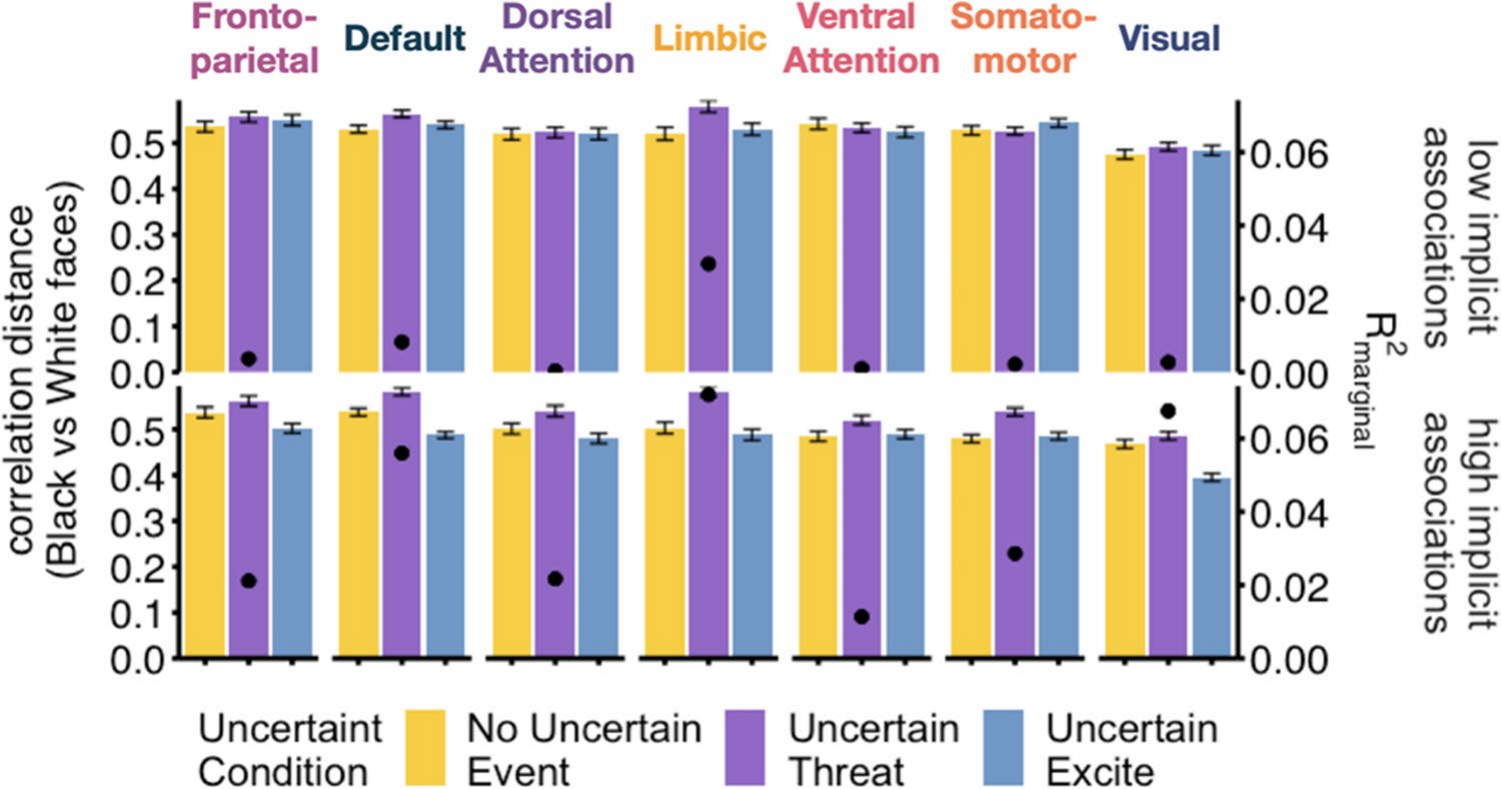
Association between neural dissimilarity to Black versus White faces for each uncertainty condition as a function of implicit racial bias. For visualization purposes, neural dissimilarity (correlation distance) to Black versus White faces by uncertainty condition is presented separately for high and low implicit racial bias groups defined by a median split on the implicit association score. Black dots represent the effect size of the uncertainty condition on correlation distance. Error bars represent 95% confidence intervals

The secondary model, including IAT scores, revealed main effects of uncertainty condition (*F*(2, 1940.00) = 7.54, *p* < 0.001), network (*F*(6, 1940.00) = 6.03, *p* < 0.001), and an interaction between uncertainty condition and IAT scores (*F*(2, 1940.00) = 5.07, *p* = 0.0064). Specifically, there was a positive association between IAT scores and correlation distance between Black and White faces during the uncertain threat condition (B = 0.0079, SE = 0.0378) and the no uncertain event condition (B = 0.036, SE = 0.0378). A negative association (B = -0.044, SE = 0.0378) was observed between IAT scores and correlation distances in the uncertain reward condition. To visualize this interaction, participants were grouped into high and low implicit bias groups based on a median split of IAT scores. Marginal R-squared values were calculated for the effect of uncertainty condition on correlation distance within each network. This revealed that participants with the strongest negative associations with Black relative to White faces showed the greatest neural dissimilarity between Black and White faces during uncertain threat across all functional networks (Fig. 4).

## Discussion

The current study used a community sample with equal representation of Black and White individuals to examine the effects of uncertain threat and race information on patterns of impulse control and brain activity. Using a psychophysiologically validated paradigm (Cohen et al., 2016) to experimentally manipulate conditions of uncertainty, we found that both Black and White individuals showed increased impulsive actions to Black compared with White faces under uncertain threat, relative to uncertain reward or no uncertain event. Paralleling our behavioral findings, Black and White faces showed higher neural dissimilarity under uncertain threat, relative to uncertain reward or no uncertain event across all functionally defined brain networks (Yeo et al., 2011) in both Black and White participants. Greater neural dissimilarity in the threat state was associated with stronger implicit racial bias. The similar neural response to Black faces in both Black and White participants provides evidence for the role of uncertain threat in response to Black faces.

The current behavioral findings add to a growing literature that suggests that exposure to Black faces can impair cognitive control processes and are consistent with findings of heightened behavioral reactivity to Black relative to White racial cues (Amodio et al., 2008; Correll et al., 2002; Payne, 2001; Richeson et al., 2003; Rubien-Thomas et al., 2021). This study extends our knowledge of race-based reactivity to cues by experimentally manipulating uncertain threat and demonstrating that impulse control to Black faces is greatest under conditions of uncertain threat compared with conditions of no uncertainty or uncertain reward. Critically, the inclusion of both no uncertain event and uncertainty of a positive event allows us to dissociate effects driven by valence (threat and reward) versus uncertainty (absence or presence of uncertain event). The robust effect of uncertain threat on brain and behavior complements previous work demonstrating diminished cognitive control under states of acute threat and stress (Cohen et al., 2016; Liston et al., 2009; Pessoa et al., 2012;Verbruggen & De Houwer, 2007). Given the effects of uncertain threat were specifically associated with greater impulsivity to Black versus White faces for both Black and White participants suggests that culturally shared racial stereotypes of Black individuals as dangerous and threatening are likely involved, rather than some general racial animus. As such automatic racial associations that are deeply ingrained in society are likely held by both Black and White participants.

Our representational similarity analysis aligned with our behavioral analyses by measuring differences in the brain’s spatial representation of Black and White faces under conditions of uncertain threat, uncertain reward and no uncertain event. Paralleling the behavioral findings of greater false alarms to Black versus White faces under uncertain threat, we observed greater neural dissimilarity in this condition, relative to comparison conditions of uncertain reward and no uncertain event across functional brain networks. In other words, the representation of Black and White faces was most discrete under uncertain threat. In contrast to previous work using univariate analyses and focusing on brain regions associated with specific functions, the current study took a functional network approach (Cole et al., 2014) to examine broad reaching differences in race processing across the brain under uncertain threat. Our analyses allowed us to compare the effects of conditions of uncertainty on the representation of race information across brain networks and postulate how relative differences might be related to the networks’ functionally attributed roles. The effect sizes of uncertainty conditions on neural dissimilarity varied across all functional networks, with the greatest effects observed in limbic, visual, and default mode networks. These findings are consistent with evidence of multiple brain networks contributing independently and together when processing or reacting to salient race-relevant information (Amodio & Cikara, 2021; Kubota, Banaji, & Phelps, 2012). Moreover, these effects were enhanced in individuals with higher levels of pro-White implicit racial bias, especially in the limbic and visual networks.

The robust effect of uncertain threat in the limbic network falls in line with previous work showing engagement of fear related circuitry in processing of both emotional information (LeDoux & Phelps, 2008; Mattavelli et al., 2014) and race information (Cunningham et al., 2004; Hart et al., 2000; Lieberman et al., 2005; Phelps et al., 2000; Richeson et al., 2008). Our results indicate that uncertain threat amplifies the baseline neural discrimination of racial group information in fear-related circuitry. Previous functional connectivity studies demonstrate that activity in the limbic network can dynamically interact with other networks critical for cognitive control (Chanes & Barrett, 2016; Lee & Telzer, 2016) and is altered when individuals experience perceived threat (Gold et al., 2015). In light of these findings, our results may suggest that, in threatening contexts, the more dissociable representation of Black and White faces in the limbic network dynamically interacts with other networks, resulting in diminished impulse control to Black faces.

The visual network is key in extracting the physical cues that differentiate faces from one another (Kanwisher & Yovel, 2006). This network showed high neural dissimilarity in the representation of Black and White faces under uncertain threat. This finding dovetails with previous work showing that experimental manipulation of external contexts (e.g., motivational states, emotional states, processing goals) can alter the engagement of the visual network in response to race (Cunningham et al., 2012; Kaul et al., 2014; Ofan et al., 2011). Specifically, we demonstrate that uncertain threat is associated with more dissociable representation of Black and White faces in the visual network, suggesting that uncertain threat alters how we visually process faces of different races—at least the faces of racial groups for which threat is a relevant social stereotype.

High neural dissimilarity under uncertain threat also was observed in the default mode network. This finding complements previous work implicating the default mode network in the processing of in-group relationships (Rilling et al., 2008) in that the categorization of racial in-group members necessitates distinct neural representation of racial groups. Critically, threatening situations can facilitate outgroup categorization (Miller, Maner, & Becker, 2010). In light of the effects of threat on group categorization, our results may provide preliminary evidence that uncertain threat contributes to the salience of group membership through increased neural dissimilarity between racial groups in the default mode network.

It is important to note that race information in the present study was orthogonal to the task design (i.e., irrelevant to the task goals of detecting emotional faces). We observe parallel findings of increased impulsive actions to Black faces and more dissociable representation of task-irrelevant race information in functional networks under uncertain threat. Our findings support the notion that race information interferes with goal-directed behavior when individuals anticipate uncertain threat. Previous work shows that negative implicit attitudes toward Black people are associated with altered frontoparietal activity (Brosch et al., 2013; Krill & Platek, 2009; Phelps et al., 2000; Rubien-Thomas et al., 2021) and diminished cognitive control (Richeson et al., 2003; Rubien-Thomas et al., 2021) upon exposure to Black faces. Accordingly, implicit bias may contribute to interference with goal-directed processing in the presence of race information related to stereotype-based threat. We demonstrate a positive association between pro-White implicit racial bias and neural dissimilarity of Black and White faces under uncertain threat that is particularly pronounced in visual and limbic networks. This finding is consistent with a framework where individuals with stronger implicit racial biases have greater interference from race information on impulse control when processing Black faces when experiencing uncertain threat.

Although Black and White participants differed in their implicit bias scores, there was overlap in the distribution of these scores for the two groups. As such, our findings are consistent with prior evidence for the evaluation of Black individuals as dangerous and threatening as a shared cultural stereotype in the United States (Eberhardt et al., 2004; Xie et al., 2021) that may influence both Black and White Americans’ evaluations. Moreover, our findings complement recent work, suggesting the role of experiences in shaping trait evaluations from faces (Chua & Freeman, 2022; Dotsch et al., 2016; Stolier et al., 2020; Stolier, Hehman, Keller, Walker, & Freeman, 2018), rather than an in-group/out-group innate mechanism related to survival. That said, we cannot say with certainty that the greater impulsivity to Black faces and greater representational dissimilarity of Black and White faces under uncertain threat are due to the same factors for Black and White participants.

It is important to note that the moderating effect of implicit bias was observed in the neural data but not in the behavioral data. Our neural findings are consistent with past work suggesting the role of implicit racial attitudes and beliefs in shaping neural activity to race-relevant stimuli (Donders et al., 2008; Hatzenbuehler et al., 2021; Krill & Platek, 2009; Phelps et al., 2000). There are several reasons why implicit bias may not have shown predictive validity in the behavioral data. It is possible that greater statistical power is necessary to observe the effects of implicit bias on behavior in this specific paradigm or that a more varied distribution of implicit bias scores is necessary to observe the effects of implicit attitudes on behavior in this specific paradigm. Future research (e.g., measuring individual experiential factors; experimentally manipulating learning counter to cultural stereotypes; assessing and quantifying participants perceptions about the study) would help to further constrain our interpretations of the absence of moderating effects of implicit bias on impulsivity to Black faces and the relationship between greater impulsivity to Black faces and greater representational dissimilarity of Black and White faces under uncertain threat.

Potential limitations of the current study should be considered. Although the manipulation of uncertainty in this paradigm has been validated previously using both psychophysiology and self-report (Cohen et al., 2016), we did not acquire these measures in the present study. As such, we cannot assure the same degree of arousal for the uncertain threat and uncertain reward conditions nor whether the uncertainty manipulations for the different valanced information (uncertain threat and uncertain reward) were temporally contained to a given block in the task. However, the observed patterns in behavior between the uncertain threat and control conditions are consistent with prior work showing diminished performance under conditions of threat relative to neutral and positive ones (Cohen et al., 2016; Liston et al., 2009). Second, the current task included emotional faces as stimuli, which could have contributed to the general arousal across the three emotional states. Although stimulus emotion did not significantly contribute to our model fits, we cannot ensure that the stimuli did not contaminate our nonarousal control state and/or impact our uncertainty manipulations. Finally, while the current study included a between-subject design with equal representation of Black and White participants, the sample is not representative of the general population of the United States. Although the sample reflects a community sample of more than 100 individuals from two different metropolitan areas and states, future work would benefit from a larger sample from urban and rural areas across the United States with equal distributions in income, education and other social factors across racial groups.

## Conclusions

In the current study, we demonstrated that individuals, regardless of their own race, show increased impulsive actions and greater dissimilarity in the neural representation of Black, compared with White, faces under uncertain threat. This study, unlike prior studies, experimentally manipulated uncertain threat to examine the role of threat in impulsive actions toward racial cues. The use of no uncertainty and uncertain reward conditions in the current go/no-go paradigm allowed for dissociation of the effects of valence (reward and threat) and uncertainty on brain and behavior and showed specificity of enhanced effects under uncertain threat. These findings highlight the potential contribution of implicit racial bias to greater neural dissimilarity of race information that may help to explain impulsive behavior in racial interactions. Critically, we provide evidence for the role of uncertain threat—rather than outgroup derogation or hostility— in the emergence of patterns of cognitive control failure in response to Black cues found in previous work (Correll et al., 2002; Richeson et al., 2003; Rubien-Thomas et al., 2021). Together, our results illustrate the distinct and important influence uncertain threat has on how some racial groups are spatially represented across the brain and how uncertain threat may contribute to racially biased behavior.

## Supplementary Information

ESM 1 (DOCX 133 kb)

## Data Availability

Data made available upon request.
