# Supplementary material for: Uncertain threat is associated with greater impulsive actions and neural dissimilarity to Black versus White faces
Source: Cogn Affect Behav Neurosci. 2023 Feb 2;23(3):944–56. doi: 10.3758/s13415-022-01056-2 (PMC10390611; doi:10.3758/s13415-022-01056-2)
Supplement: Supplementary file 1 — (DOCX 133 kb) [file 13415_2022_1056_MOESM1_ESM.docx]

Supplemental Figures


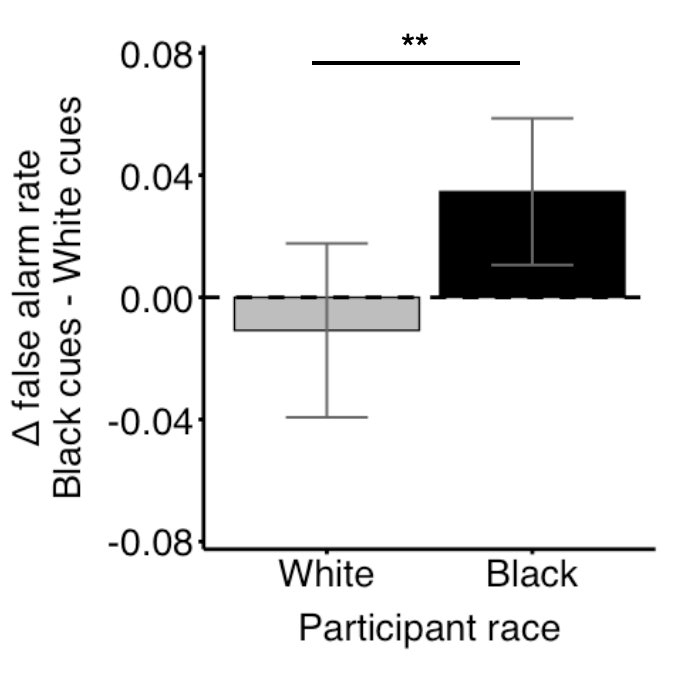


Figure S1. Visualization of participant race x stimulus race interaction predicting false alarm rates. Black participants make more false alarms to Black versus White faces compared to White participants. Error bars represent 95% confidence intervals.


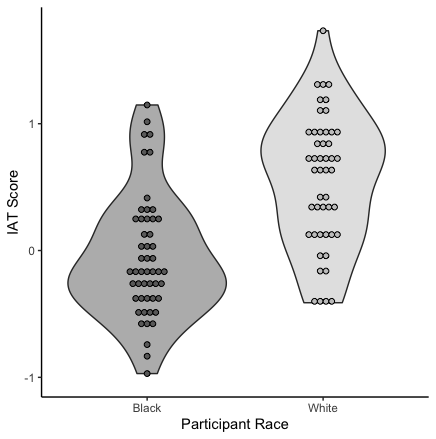


Figure S2. Distribution of Implicit Association Test scores for Black and White participants.

Table S1. Linear mixed effects analysis comparing models predicting false alarm rates with and without a stimulus race x uncertainty condition interaction.

|  | | |
| --- | --- | --- |
|  | *Dependent variable:* | |
|  | False alarm rate - centered  Beta value (Standard Error) | |
|  | Full model | Null interaction model |
| Stimulus Race | -0.050 | 0.055 |
|  | (0.062) | (0.036) |
|  |  |  |
| Uncertainty condition (Reward) | -0.046 | -0.046 |
|  | (0.044) | (0.044) |
|  |  |  |
| Uncertainty condition (Threat) | **0.134^**^** | **0.134^**^** |
|  | **(0.044)** | **(0.044)** |
|  |  |  |
| Participant race | -0.033 | -0.033 |
|  | (0.176) | (0.176) |
|  |  |  |
| Participant gender | **-0.528^**^** | **-0.528^**^** |
|  | **(0.169)** | **(0.169)** |
|  |  |  |
| Stimulus race x Uncertainty condition (Reward) | 0.070 |  |
|  | (0.088) |  |
|  |  |  |
| Stimulus Race x Uncertainty condition (Threat) | **0.245^**^** |  |
|  | **(0.088)** |  |
|  |  |  |
| Participant race x Uncertainty condition (Reward) | **0.290^*^** | **0.212^**^** |
|  | **(0.124)** | **(0.072)** |
|  |  |  |
| Stimulus race x Participant race | 0.091 | 0.091 |
|  | (0.088) | (0.089) |
|  |  |  |
| Participant race x Uncertainty condition (Threat) | -0.120 | -0.120 |
|  | (0.088) | (0.089) |
|  |  |  |
| Participant race x Stimulus race x Uncertainty condition (Reward) | -0.210 |  |
|  | (0.175) |  |
|  |  |  |
| Participant race x Stimulus race x Uncertainty condition (Threat) | -0.023 |  |
|  | (0.175) |  |
|  |  |  |
| constant | -0.017 | -0.017 |
|  | (0.088) | (0.088) |
|  |  |  |
| Observations | 630 | 630 |
| Log Likelihood | -552.107 | -557.106 |
| Akaike Inf. Crit. | 1,138.215 | 1,140.211 |
| Bayesian Inf. Crit. | 1,213.792 | 1,198.006 |
|  | | |
|  | ^*^p<0.05; ^**^p<0.01; ^***^p<0.001 | |

*Note: White stimuli, the no uncertain event condition, White participants, and male participants are reference groups.*

Table S2. Summary of False Alarm Rates by Stimulus Race and Uncertainty Condition

| Stimulus Race | Condition | Mean False Alarm Rate | Standard Deviation |
| --- | --- | --- | --- |
| Black | No Uncertain Event | 0.3157407 | 0.08752737 |
| White | No Uncertain Event | 0.3378307 | 0.09699766 |
| Black | Uncertain Reward | 0.3133598 | 0.09289379 |
| White | Uncertain Reward | 0.3215608 | 0.09557102 |
| Black | Uncertain Threat | 0.3701058 | 0.09110608 |
| White | Uncertain Threat | 0.3398148 | 0.08951791 |

Table S3. Linear mixed effects analysis comparing models predicting correlation distance with and without a factor of uncertainty condition

|  | | | |
| --- | --- | --- | --- |
|  | *Dependent variable:* | | |
|  | Correlation distance  Beta value (Standard Error) | | |
|  | Full model | Null state model |  |
| Uncertainty condition (Reward) | 0.012 |  |  |
|  | (0.022) |  |  |
| Uncertainty condition (Threat) | 0.031 |  |  |
|  | (0.022) |  |  |
| Network (Frontoparietal) | 0.032 | 0.024 |  |
|  | (0.022) | (0.013) |  |
| Network (Default) | 0.030 | 0.023 |  |
|  | (0.022) | (0.013) |  |
| Network (Dorsal Attention) | 0.006 | -0.003 |  |
|  | (0.022) | (0.013) |  |
| Network (Limbic) | 0.007 | 0.017 |  |
|  | (0.022) | (0.013) |  |
| Network (Salience/Ventral Attention) | 0.009 | -0.001 |  |
|  | (0.022) | (0.013) |  |
| Network (Visual) | -0.033 | **-0.050^***^** |  |
|  | (0.022) | **(0.013)** |  |
| Uncertainty condition (Reward) x Network (Frontoparietal) | -0.020 |  |  |
|  | (0.031) |  |  |
| Uncertainty condition (Threat) x Network (Frontoparietal) | -0.005 |  |  |
|  | (0.031) |  |  |
| Uncertainty condition Reward) x Network (Default) | -0.030 |  |  |
|  | (0.031) |  |  |
| Uncertainty condition (Threat) x Network (Default) | 0.010 |  |  |
|  | (0.031) |  |  |
| Uncertainty condition (Reward) x Network (Dorsal Attention) | -0.020 |  |  |
|  | (0.031) |  |  |
| Uncertainty condition (Threat) x Network (Dorsal Attention) | -0.007 |  |  |
|  | (0.031) |  |  |
| Uncertainty condition (Reward) x Network (Limbic) | -0.013 |  |  |
|  | (0.031) |  |  |
| Uncertainty condition (Threat) x Network (Limbic) | 0.042 |  |  |
|  | (0.031) |  |  |
| Uncertainty condition (Reward) x Network (Salience/Ventral Attention) | -0.016 |  |  |
|  | (0.031) |  |  |
| Uncertainty condition (Threat) x Network (Salience/Ventral Attention) | -0.015 |  |  |
|  | (0.031) |  |  |
| Uncertainty condition (Reward) x Network (Visual) | -0.041 |  |  |
|  | (0.031) |  |  |
|  |  |  |  |
| Uncertainty condition (Threat) x Network (Visual) | -0.011 |  |  |
|  | (0.031) |  |  |
| Constant | 0.502^***^ | **0.516^***^** |  |
|  | (0.017) | **(0.011)** |  |
| Observations | 2,079 | 2,079 |  |
| Log Likelihood | 853.174 | 835.753 |  |
| Akaike Inf. Crit. | -1,656.348 | -1,649.506 |  |
| Bayesian Inf. Crit. | -1,515.357 | -1,587.470 |  |
|  | | | |
|  | ^*^p<0.05; ^**^p<0.01; ^***^p<0.001 | | |

*Note: The no uncertain event condition and Somatomotor network are reference groups.*
